# Transcatheter tricuspid valve replacement: will it prevail?

**DOI:** 10.3389/fcvm.2025.1562658

**Published:** 2025-04-16

**Authors:** Xiling Zhang, Nina Sophie Pommert, David Meier, Stephanie L. Sellers, Hatim Seoudy, Oliver J. Müller, Derk Frank, Tim Attmann, Rouven Berndt, Gregor Warnecke, Thomas Puehler, Georg Lutter

**Affiliations:** ^1^ Department of Cardiac Surgery, University Hospital Schleswig-Holstein (UKSH), Kiel, Germany; ^2^ DZHK (German Centre for Cardiovascular Research), Partner Site Hamburg/Kiel/Lübeck, Hamburg, Germany; ^3^ Department of Cardiac and Thoracic Vascular Surgery, University Hospital Schleswig-Holstein (UKSH), Luebeck, Germany; ^4^ Department of Cardiology, Lausanne University Hospital and University of Lausanne, Lausanne, Switzerland; ^5^ Centre for Cardiovascular Innovation, St Paul’s and Vancouver General Hospital, Vancouver, BC, Canada; ^6^ Cardiovascular Translational Laboratory, Providence Research & Centre for Heart Lung Innovation, Vancouver, BC, Canada; ^7^ Centre for Heart Valve Innovation, St. Paul’s Hospital, University of British Columbia, Vancouver, BC, Canada; ^8^ Department of Cardiology and Angiology, University Hospital Schleswig-Holstein (UKSH), Kiel, Germany; ^9^ Department of Vascular Surgery, University Hospital Schleswig-Holstein (UKSH), Kiel, Germany

**Keywords:** tricuspid regurgitation, heart valve replacement, transcatheter, stents, clinical outcomes

## Abstract

Severe tricuspid regurgitation (TR) is a prevalent and challenging condition associated with poor survival outcomes and significant morbidity. Medical therapy alone often fails to provide adequate symptom relief, and stand-alone surgical intervention is linked to high mortality rates, making it a less favorable option unless combined with left-sided valve surgery. The advent of transcatheter tricuspid interventions has provided new therapeutic possibilities, particularly for high-risk patients who are ineligible for conventional surgery. However, many patients are still unsuited for transcatheter tricuspid repair or have only limited benefits from such procedures. In this context, Transcatheter tricuspid valve replacement (TTVR) has rapidly emerged as a promising alternative, offering the potential for more effective treatment outcomes. This review explores the latest advancements in TTVR devices, highlights key clinical findings, and discusses the challenges and limitations of this evolving strategy. Additionally, we address patient selection criteria, procedural outcomes, and future directions in the field, emphasizing the potential of TTVR to transform the management of severe TR.

## Introduction

Tricuspid regurgitation (TR) is a prevalent form of valvular heart disease, with extensive research establishing its severity as an independent predictor of mortality (1). For patients with significant TR, timely intervention is crucial to preventing right ventricular (RV) dilatation and dysfunction (2, 3). Both American [American College of Cardiology (ACC)/American Heart Association (AHA)] (4) and European [European Society of Cardiology (ESC)/European Association for Cardio-Thoracic Surgery (EACTS)] guidelines recommend surgery in patients with severe TR undergoing left-sided valve surgery (Class I) (2). Additionally, they advocate tricuspid valve (TV) surgery in patients with mild-to-moderate secondary TR undergoing left-sided valve surgery if tricuspid annular dilatation or prior signs and symptoms of right-sided heart failure are present. Moreover, severe isolated secondary TR caused by annular dilation, even in the absence of pulmonary hypertension (PH) or left-sided disease, is classified as a Class IIa indication for TV surgery. In contrast, for severe isolated primary TR, the ESC/EACTS guidelines assign a Class I indication for symptomatic patients or those with signs of right-sided heart failure, whereas the ACC/AHA guidelines categorize it as Class IIa-B (5), see Table 1.

**Table 1:** International guidelines’ recommendations on management of TR.

Recommendation	ACC/AHA	ESC/EACTS
TV surgery in patients undergoing left-sided valve surgery		
Severe TR	I-B	I-B (secondary TR) or I-C (primary TR)
Moderate primary TR		IIa-C
Secondary TR and TA > 40 mm or prior signs of right-sided heart failure	IIa-B (progressive TR)	IIa-B (mild or moderate TR)
TV surgery in severe primary TR		
No or mild symptoms and RV dilatation		IIa-C
Symptoms and signs of right-sided heart failure	IIa-B	I-C (without severe RV dysfunction)
Progressive RV dilation or systolic dysfunction	IIb-C	
TV surgery in severe secondary TR		
Symptoms and RV dilatation and no severe RV or LV dysfunction or severe PH	IIb-B	IIa-C
Symptoms and signs of right-sided HF and no PH or left-sided disease or response to medical therapy	IIa-B	
Transcatheter treatment of symptomatic secondary severe TR in inoperable patients at a heart valve center with dedicated expertise		IIb-c

LV, left ventricle; PH, pulmonary hypertension; RV, right ventricle; TA, tricuspid annulus; TR, tricuspid regurgitation; TV, tricuspid valve (5).

However, managing isolated TR, particularly when accompanied by RV dysfunction, remains challenging, with perioperative mortality rates reaching up to 10% (2).

Transcatheter tricuspid valve intervention (TTVI) offers a therapeutic approach to mitigate the risks associated with conventional surgical procedures. Moreover, compared to treatment with oral medications alone, TTVI may be associated with higher survival rates and lower rates of heart failure rehospitalization (6). Recent advancements have introduced various TTVI techniques, providing minimally invasive alternatives that have shown promising initial results. The spectrum of TTVI includes both transcatheter tricuspid valve repair (TTV**r**) and replacement (TTV**R**), each tailored to specific patient needs. While TTVr has demonstrated commendable safety and efficacy, anatomical considerations such as unfavorable tricuspid valve morphology or excessive annular dilatation with a large coaptation gap may preclude the use of edge-to-edge repair. For these individuals, TTVR sometimes represents the only potential alternative (7).

This review comprehensively examines the different types of valves currently available and their respective status in clinical trials. Furthermore, the ongoing challenges and developmental trends in the field of TTVR are analyzed, emphasizing the potential of TTVR to significantly advance the therapeutic landscape for TR.

## Transcatheter tricuspid valve replacement—current landscape

Since the first in-human implantation, TTVR has advanced rapidly (8). Early devices were temporarily abandoned due to technical limitations and the complex anatomy of the tricuspid valve, which posed challenges in initial design and clinical application. However, with advancements in imaging navigation, catheter technology, and materials science, TTVR techniques have gradually improved, now encompassing two primary approaches: orthotopic and heterotopic replacement.

Orthotopic replacement involves directly implanting a new valve at the tricuspid valve site, while heterotopic replacement positions the valve stent within the vena cava (9). Multiple new devices are currently entering clinical trials, with some demonstrating significant efficacy in high-risk patients (for details see Table 2).

**Table 2:** Orthotopic and heterotopic tricuspid valve replacement devices currently under development and testing.

Device	Manufacturer	Access	Anchoring	Trials
Orthotopic				
Transfemoral				
VDyne	VDyne	Transfemoral	Septal anchor	NCT05797519
Cardiovalve	Venus MedTech	Transfemoral	TV leaflets	NCT04100720
Evoque*	Edwards Lifescience	Transfemoral	TV leaflets/annulus	NCT04221490 NCT04482062
Intrepid	Medtronic	Transfemoral	Perimeter oversizing	NCT04433065
TRiCares	TRiCares	Transfemoral	Tricuspid annulus	NCT05126030
Transatrial				
LuX-Valve	Jenscare biotechnology	Transatrial	Septal anchor and anterior leaflet grasp	NCT05436028
NaviGate	NaviGate cardiac structures	Transatrial	TV leaflets/annulus	N/A
Transjugular				
Lux-Valve Plus	Jenscare biotechnology	Transjugular	Septal anchor and anterior leaflet grasp	NCT05436028
Trisol	Trisol medical	Transjugular	Tricuspid annulus	NCT04905017
Heterotopic				
Sapien XT	Edwards Lifescience	Transfemoral	Preceding stent implantation	NCT02339974
TricValve	Products + Features	Transfemoral	N/A	NCT04141137
Tricento	MEDIRA	Transfemoral	N/A	N/A

The Evoque valved stent received CE mark*. The scaffolds of these valved stents are made of nitinol. TV, tricuspid valve.

### Orthotopic transcatheter tricuspid valve replacement

#### Transfemoral access routes

##### VDyne

**Figure 1:**
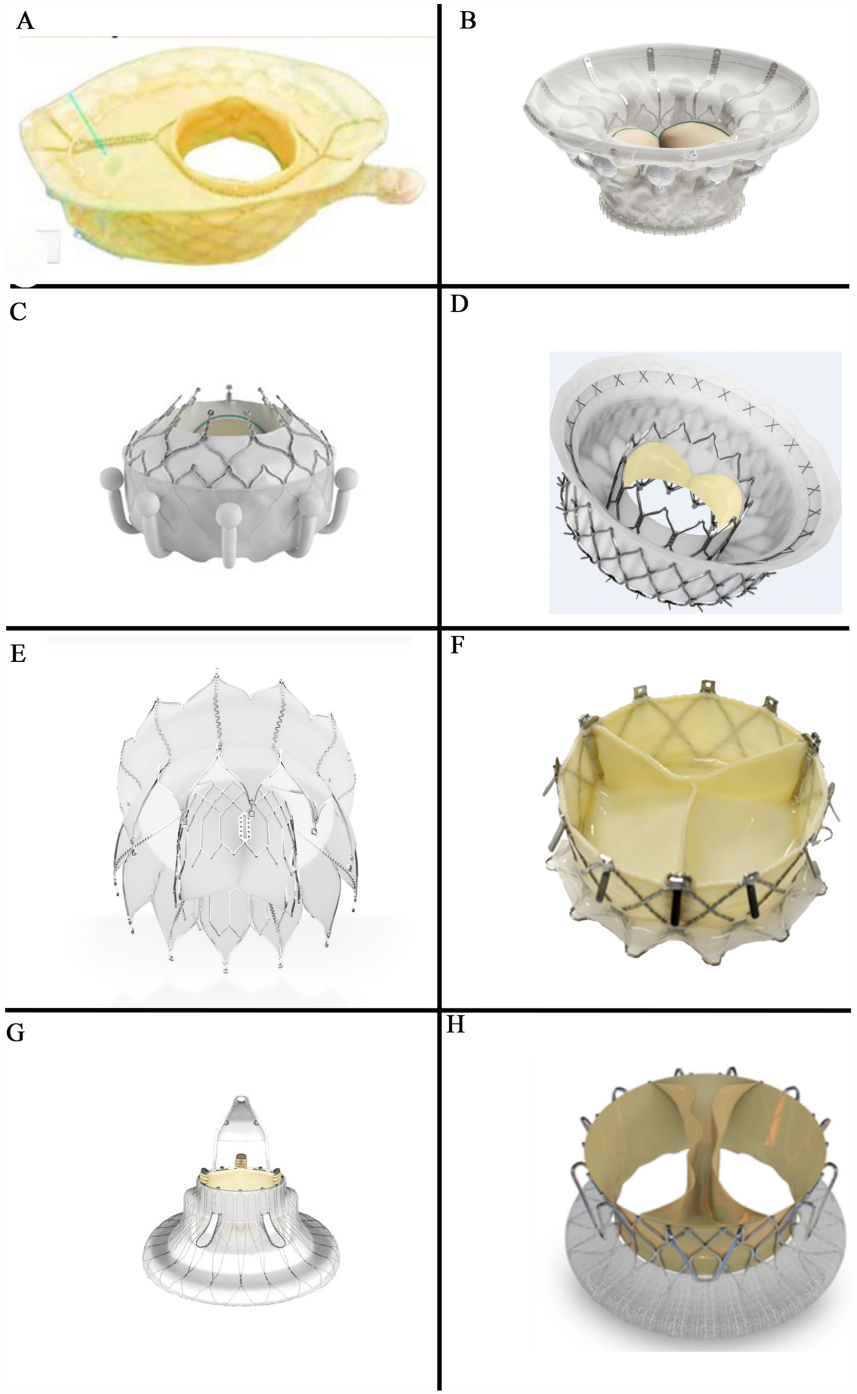
Orthotopic transcatheter valved stents: **(A)** the vDyne valve (provided by VDyne, Inc., Maple Grove, MN, USA); **(B)** the cardiovalve (provided by Venus MedTech, Hangzhou, China); **(C)** the EVOQUE (provided by Edwards Lifesciences, Irvine, CA, USA); **(D)** the intrepid valve (provided by Medtronic Plc, MN, USA); **(E)** the tRiCares topaz valve (provided by TRiCares SAS, Paris, France); **(F)** the naviGate valve (provided by NaviGate Cardiac Structures Inc., Lake Forest, CA, USA); **(G)** the LuX-valve (provided by Jenscare Scientific Co., Ltd, Ningbo, China); **(H)** the trisol valve (provided by Trisol Medical, Yokneam, Israel).

The VDyne valve (VDyne, Inc., Maple Grove, MN, USA, Figure 1A) consists of a dual-frame nitinol prosthesis, housing a 30 mm porcine tri-leaflet valve. The outer frame is asymmetrically designed (like an oyster, pear-like) with five different fixation mechanisms: a tab at the RV outflow tract, small tabs at the lateral or free wall of the RV, a small tab at the posteroseptal wall, and a large tab beyond the posterior annulus. This design aims to anatomically conform to the native annulus while allowing for minor oversizing (larger atrial and ventricular hub). The valve is available in seven sizes and is suitable for tricuspid annuli with circumferences of up to 180 mm—additional sizes are reportedly under development. The valve is deployed using a single 28Fr catheter with a side-loading delivery system in which the prosthesis is crimped vertically rather than radially. After full expansion and positioning, it can be fully recaptured (10).

The initial thirteen patients receiving the 3rd generation of human implants were all successfully treated (10). Early feasibility trials are currently underway in multiple regions globally (VISTA, NCT05797519), and the device has been designated as a breakthrough device by the U.S. Food and Drug Administration (FDA).

This device relies on five different fixation mechanisms rather than a uniform circular support like Evoque (two circular plates) because the circular shape does not fill out the commissural corners and paravalvular leakage (PVL) occurs. In contrast, the oyster shape of VDyne more optimally avoids paravalvular leakages due to anatomical shape mimicry and multiple anchoring systems. Although the design may better accommodate the anatomy of the tricuspid annulus, its asymmetrical structure might render it unsuitable for some patients.

The side-loading delivery method improves valve deployment by accommodating larger valve sizes compared to circular-loading systems (e.g., Evoque, Lux-valve). However, its implementation requires operators to adapt to different procedural steps. A significant advantage compared to all other devices is that the operator can retrieve the TTVR valved stent after full deployment.

##### Cardiovalve

The Cardiovalve (Venus MedTech, Hangzhou, China, Figure 1B) device comprises a self-expanding nitinol stent and bovine pericardial leaflets. It features an atrial flange to assist with anchoring and incorporates leaflet capture technology to prevent valve migration. The valve is delivered via a low-profile 28F delivery system through the femoral vein. The Cardiovalve is suitable for patients with an annulus diameter ranging from 36 to 55 mm and a RV length exceeding 45 mm (11).

An early feasibility study of the Cardiovalve with 15 patients conducted in the United States (NCT04100720) has been temporaritly interrupted due to technical issues. The primary endpoints include the absence of device- or procedure-related adverse events within 30 days post-procedure. The new TARGET trial to evaluate the safety and performance of the Cardiovalve system (NCT05486832) on an expected 100 patients, has commenced.

This device requires a certain RV length (>45 mm), limiting its use in patients with a shorter RV length. Moreover, its long-term durability and safety have not yet been fully validated.

A pancake-like fixation design (two circular plates) might lead to insufficient stability of the valve in certain anatomical structures, increasing the risk of displacement, PVL, or rotation.

##### Evoque

The EVOQUE tricuspid valve replacement system (Edwards Lifesciences, Irvine, CA, USA, Figure 1C) includes a self-expanding nitinol stent, bovine pericardial leaflets, an intra-annular big sealing skirt, and nine ventricular anchoring devices. The three available sizes are: 44 mm, 48 mm, and 52 mm. It utilizes a low-profile, multi-plane 28Fr delivery system designed for femoral artery implantation, making it adaptable to a wide range of anatomical structures.

The Evoque device has been used very often in the mitral position to reduce mitral regurgitation. It has also been used in the tricuspid position, but in the mitral position the device proved unsuccessful.

The recent TRISCEND trial (NCT04221490) on the tricuspid valve evaluated the safety and performance of the EVOQUE system in patients with symptomatic TR of at least moderate severity despite having received medical therapy. At 1 year, 97.6% of implanted patients had TR of mild or less, with 69.0% exhibiting none or only trace TR (12).

The TRISCEND II trial (NCT04482062) assessed the safety and efficacy of the EVOQUE system compared to guideline-directed medical therapy (GDMT) for patients with at least severe TR. Initial six-month follow-up results from the first 150 patients demonstrated that the EVOQUE system effectively eliminated TR in approximately 78% of participants, with nearly 99% achieving a TR severity of moderate or less and about 94% reaching a severity of mild or less (13). Results from the second-phase TRISCEND II trial, involving 400 patients, indicated that at 1 year, 72.6% of patients had no residual TR, 22.6% had mild regurgitation, 3.8% had moderate regurgitation, and 0.9% had severe regurgitation. Although most patients experienced a significant reduction in regurgitation severity, the incidence of important safety outcomes also warrants attention, including 30-day all-cause mortality (3.5%), major bleeding within 30 days (10.4%), and new pacemaker implantation within 1 year (17.8%) (14). The EVOQUE tricuspid valve replacement system has received Conformité Européene (CE) certification from the European Union (2023) and approval from the U.S. FDA (2024).

The intra-annular sealing skirt of this device might reduce PVL and improve hemodynamic stability because the large intra-annular sealing skirt enhances the conformity between the prosthetic valve and the native annulus. Our Kiel experience with one case demonstrated that you can also implant this device using the jugular approach.

Additionally, the device has received CE certification and FDA approval. However, results of clinical trials indicate a relatively high postoperative pacemaker implantation rate (17.8%), which might influence device selection for certain patients.

This pancake-like fixation design (two circular plates) might lead to insufficient stability of the valve in certain anatomical structures.

##### Intrepid

The Intrepid valve (Medtronic Plc, Minneapolis, MN, USA, Figure 1D) is specifically designed for patients with mitral regurgitation (MR) and now is also for TR. In the mitral position the Intrepid has been implanted in more than 600 patients worldwide.

The Intrepid valve employs a sophisticated dual-structure design (stent in stent) and was primarily used in the mitral position. This includes a 29 mm tri-leaflet bovine pericardial valve and is available in three sizes: 43 mm, 46 mm, and 50 mm. The device is currently deployed via a 35Fr delivery system accessed through the femoral vein, with ongoing further enhancements in a 29Fr system.

Following transcatheter mitral valve replacement using the transapical Intrepid valve, 99.5% (*n* = 200) of patients had mild or less MR within 30 days, and all surviving patients (*n* = 122) had mild or less MR at the 2-year follow-up (15). A pre-market trial is currently underway (APOLLO, NCT03242642). Moreover, successful cases of compassionate use have been documented (16). The first three trans-septal TMVR cases have been performed in Canada this year.

The early feasibility trial of the Intrepid TTVR (NCT04433065) is actively enrolling participants. This ongoing trial evaluates the valve's safety and efficacy in clinical settings.

The Intrepid valve was originally designed for MR and has only recently been adapted for TR. This suggests that its anatomical compatibility may require further evaluation, and its ability to achieve stable adaptation to the tricuspid anatomy remains uncertain.

##### Tricares topaz system

The TRiCares Topaz TTVR system (TRiCares SAS, Paris, France, Figure 1E) utilizes a self-expanding dual-stent design (stent in stent) made from nitinol. The outer stent provides robust sealing and anchorage while protecting the inner stent from deformation caused by RV contractions. This inner stent houses an independent porcine pericardial trileaflet valve, protecting the valve's integrity from the external stent's movements. The device is delivered through a 29Fr system accessed via the femoral vein.

The inaugural human implantation of the TRiCares Topaz system was conducted for compassionate use in treating TR and successfully implanted in two patients. During the three-month follow-up, there were no reported mortalities or complications. However, a decline in RV function was observed three months post-implantation (17). This might be due to an acute increase in afterload caused by the reduction of postoperative TR after TTVR. This can also happen after TTVR with other devices. If the RV function is pre-operatively low, it is more likely to happen.

A pioneering clinical trial of the TRiCares Topaz trans-femoral tricuspid heart valve replacement system (TRICURE, NCT05126030) is ongoing, focusing on evaluating its preliminary safety and efficacy parameters.

#### Transatrial access routes

##### Navigate

The NaviGate valve (NaviGate Cardiac Structures Inc., Lake Forest, CA, USA, Figure 1F) was the first TTVR stent implanted in humans worldwide. It has a self-expanding, tapered nitinol stent with tri-leaflet bovine pericardial leaflets. It secures the tricuspid valve using 12 anchoring screws and stabilizes with 12 atrial flaps. Available in six sizes ranging from 36 to 54 mm, the delivery system uses a 42Fr catheter sheath for transatrial or transjugular implantation. However, the transjugular approach has been abandoned due to the sheath size and complications, such as difficulty in achieving coaxial alignment with the relatively simple delivery system (11).

Navia et al. (8) reported the first use of the NaviGate valve in two patients with TR, demonstrating its safety and feasibility. In a report on 32 patients treated with NaviGate under compassionate use, the implant success rate was 100%, with all patients achieving TR severity of grade 2 or less. The 30-day mortality rate was 12.5% (18). In an early multicenter experience involving 30 patients, 26 (87%) had successful procedures, with 4 cases of device dislocation and 2 patients (5%) requiring conversion to open-heart surgery. The results of 24 patients, 18 (76%) showed mild or less TR at discharge. The in-hospital mortality rate was 10%, and 4 patients (13%) died during follow-up, with an average follow-up duration of 127 ± 82 days (19).

Due to the flexibility and anatomical variability of the tricuspid annulus, excessive reliance on screw fixation may lead to uneven distribution of forces, increasing the risk of valve dislodgement or displacement. The 30-day postoperative mortality rate (12.5%) was significantly higher compared, for instance, to that of Evoque (3.5%). Further NaviGate valve development is on hold.

#### Transjugular access routes

##### LuX-Valve

The LuX-Valve (Jenscare Biotechnology, Ningbo, China, Figure 1G) is a self-expanding bovine pericardial valve mounted on a nitinol stent. Its anchoring mechanism differs from traditional stent devices, by securing placement through anterior leaflet clamps and a ventricular anchor and significantly reducing stress on the cardiac walls and minimizing the risk of complications.

This bioprosthesis is available in four sizes, ranging from 30 to 55 mm, and includes eight skirted atrial disc options, for compatibility with native tricuspid annulus diameters from 25 to 50 mm. Implantation by a flexible 32Fr delivery system via a transatrial approach, in order to improve procedural adaptability and patient recovery (20).

In clinical evaluations, Lu et al. (20) documented the first deployment of the LuX-Valve for transcatheter tricuspid valve replacement in patients at high risk for TR. The procedure was successful in all 12 patients, with 90.9% exhibiting no residual TR at the 30-day postoperative follow-up.

Additionally, Sun et al. (21) observed a significant reduction in TR severity over 12 months in a similar patient cohort, although one patient succumbed to right heart failure within three months post-operation.

The second-generation LuX-Valve is transitioning to a transjugular approach. The first-in-human study of the LuX-Valve Plus demonstrated good results with none/trace TR within the 30 days (22). Results from 76 patients under early compassionate use showed that at 1 month, 95.0% of patients had TR of ≤2+, and 86.8% had TR of ≤1+ (23).

Multiple studies (NCT06568003, NCT05436028) are currently evaluating the safety and efficacy of transjugular tricuspid valve replacement using the LuX-Valve Plus system.

The Lux-Valve is suitable for tricuspid annulus diameters ranging from 25 to 50 mm, making it more favorable for patients with a smaller annuli. In addition to the potential for a sudden increase in RV afterload postoperatively, ventricular anchoring may also affect RV function. Given that the RV wall is thinner than the left, excessive anchoring forces could restrict RV motion.

##### Trisol valve

The Trisol valve (Trisol Medical, Yokneam, Israel, Figure 1H) is constructed from a self-expanding conical nitinol alloy and includes a single-leaflet circular bovine pericardial valve leaflet. The valve employs a high closing volume design (24) and its stability is ensured by applying axial force at the ventricular end and a polyester atrial skirt, delivered via the transjugular route using a 30Fr system. The Trisol valve has already been implanted in ten human patients (25).

For an early feasibility study of the Trisol system (NCT04905017) participants are being actively recruited to evaluate its clinical efficacy and safety further.

The long-term hemodynamic adaptability of the single-leaflet design must still be validated, and its potential impact on valve durability and turbulent flow generation is still unknown. Additionally, regarding durability, a single-leaflet design may be more prone to degeneration over time compared to a trileaflet configuration. Currently, there is insufficient data to support its long-term stability.

#### Heterotopic or caval tricuspid valve implantation

In some cases, transcatheter therapy may not be feasible for certain patients. Although this method was abandoned in the past due to low efficacy and high mortality caused by technical limitations, innovations in recent years have led to the development of heterotopic or caval tricuspid valve implantation (CAVI) as a palliative alternative. This technique reduces venous reflux and improves right heart pressure through implantation in the inferior vena cava (IVC) or superior vena cava (SVC) (26, 27). However, due to the risks of embolization, thrombosis, and hepatic vein obstruction, heterotopic implantation is more challenging than orthotopic implantation. Although CAVI does not significantly improve hemodynamics, it leads to notable improvements in quality of life and symptom relief (28). Recent reports have indicated right heart reshape remodeling after CAVI (29).

##### Sapien

The balloon-expandable Sapien valve series (Edwards Lifesciences, Irvine, CA, USA) is widely used in transcatheter aortic valve replacement (TAVR). However, for the largest 29 mm Sapien XT valve, the diameter of the IVC is still too large, necessitating anchoring within a previously placed stent in the IVC.

The first human trial was conducted in 2013 and achieved acceptable results (30). The TRICAVAL trial showed improvements in NYHA classification and quality of life post-procedure. However, the high rate of valve dislocation led to some patients requiring open-heart surgery (31).

Currently, there is no available data on the safety and efficacy of the Sapien 3 valve for treating severe, refractory TR. It can be mainly used for valve-in-valve in the tricuspid position.

##### Tricvalve

**Figure 2:**
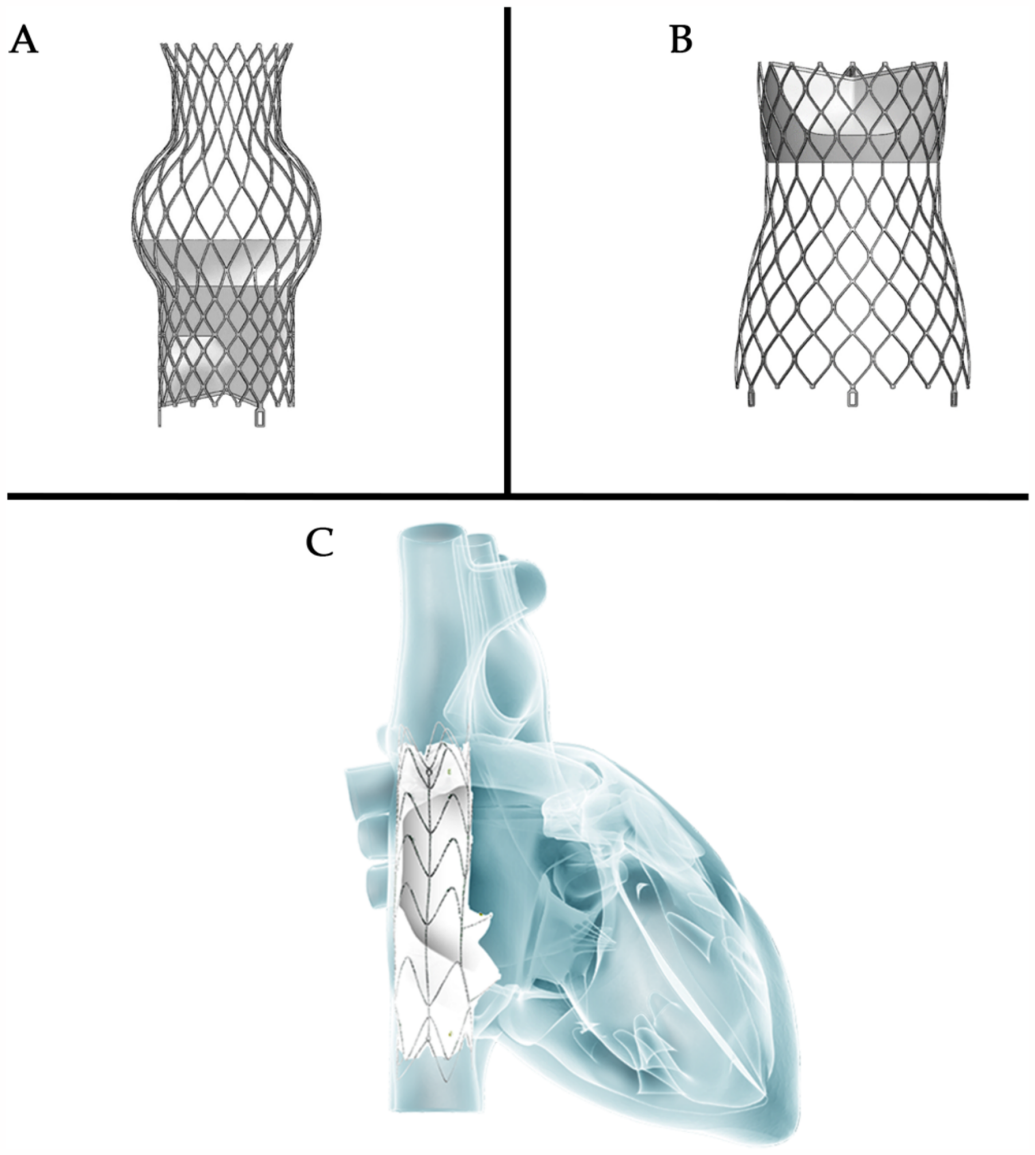
Heterotopic valve stent: **(A**,**B)** the tricValve transcatheter bicaval valve system (P + F products + features, Vienna, Austria); **(C)** the TRICENTO valved stent (Figure 2C provided by MEDIRA GmbH, Balingen, Germany).

The TricValve transcatheter bicaval valve system (Products + Features, Vienna, Austria) consists of two self-expanding nitinol stents with bovine pericardial valves. The SVC valve is available in 25 mm and 29 mm sizes with a long skirt design to prevent PVL (Figure 2A). The IVC valve, sized at 31 mm and 35 mm, has a short skirt design to prevent hepatic vein embolization (Figure 2B). The device is delivered via a 24Fr transfemoral delivery system and has received CE certification.

The first-in-human implantation of the TricValve system was successful, with an 8-week follow-up showing improvements in venous congestion and symptoms related to right heart failure (9). At 12 months post-implantation, the patient remained in NYHA class II with no symptoms of right heart failure (32). Six-months results from the TRICUS EURO study (NCT04141137) in Europe showed that 79.4% of patients were in NYHA class I or II. Prosthesis dislocation occurred in 3% of patients but did not lead to serious consequences. Major bleeding was the primary serious complication, affecting 17.1% of patients.

To observe any clinical effect, it is necessary to implant two valved stents into the two caval veins, even though hemodynamics parameters remain unchanged afterwards.

##### TRICENTO

The TRICENTO (MEDIRA, Balingen, Germany, Figure 2C) is a transcatheter bicaval valved stent currently in development, consisting of a self-expanding nitinol frame and two porcine pericardial valves. The stent is custom-made based on pre-procedural imaging of the patient and delivered via a 24Fr delivery system. It is anchored within the IVC and SVC, aligning the stent valve with the native tricuspid valve. This design is engineered to prevent systolic blood backflow and ensure forward blood flow during diastole.

Early results from a multicenter study indicated that all stents were successfully implanted; however, three cases (14%) of asymptomatic stent fractures were observed during follow-up. At a median follow-up of 61 days, 65% of patients were in NYHA class I/II. Heart failure rehospitalization occurred in 19% of patients, and the one-year survival rate was 76% (33).

A very large device which covers a moderate distance in the caval veins has to be implanted to get two valved stents fixed in the caval veins. Furthermore, one must be very sure that the opening of the device is correctly positioned towards the right atrium. Two valves might have a higher risk of degeneration than one valve.

## Current status and challenges

### Timing of intervention

For intervention in isolated TR, only the ESC/EACTS guidelines provide a Class I indication, which is for severe symptomatic TR. Wang and associates compared the characteristics and outcomes of patients with Class I indications for severe symptomatic TR to those without such indications who underwent early surgery (34). The results demonstrated significantly better short- and long-term outcomes in the early surgery group. Although the patients in Class I were older, with more pronounced symptoms and higher NYHA classifications, resulting in notable differences in baseline characteristics, the small sample size may also have influenced these findings. Nevertheless, this raises a new consideration: should we wait until Class I indications are met before intervening? In fact, the longer the wait for Class I indications, the greater the likelihood of developing risk factors such as RV dysfunction, atrial fibrillation, and renal impairment which in turn increase both surgical and long-term risks (34).

Mohamed et al. compared the outcomes of TTVI (TTVR and TTVr patients) with those of conventional surgical tricuspid valve repair (35). Their findings suggested that TTVI is associated with a lower in-hospital mortality rate and a lower incidence of cardiovascular composite complications. With the emergence and advancement of new TTVI devices, a lower-risk surgical alternative is now available. Currently, clinical experience and evidence regarding the efficacy of early TTVI remain limited. In particular, there is a lack of direct comparative studies between early TTVI and early surgical intervention. Therefore, further clinical research is required to establish the safety and efficacy of early TTVI.

### Patient selection

**Figure 3:**
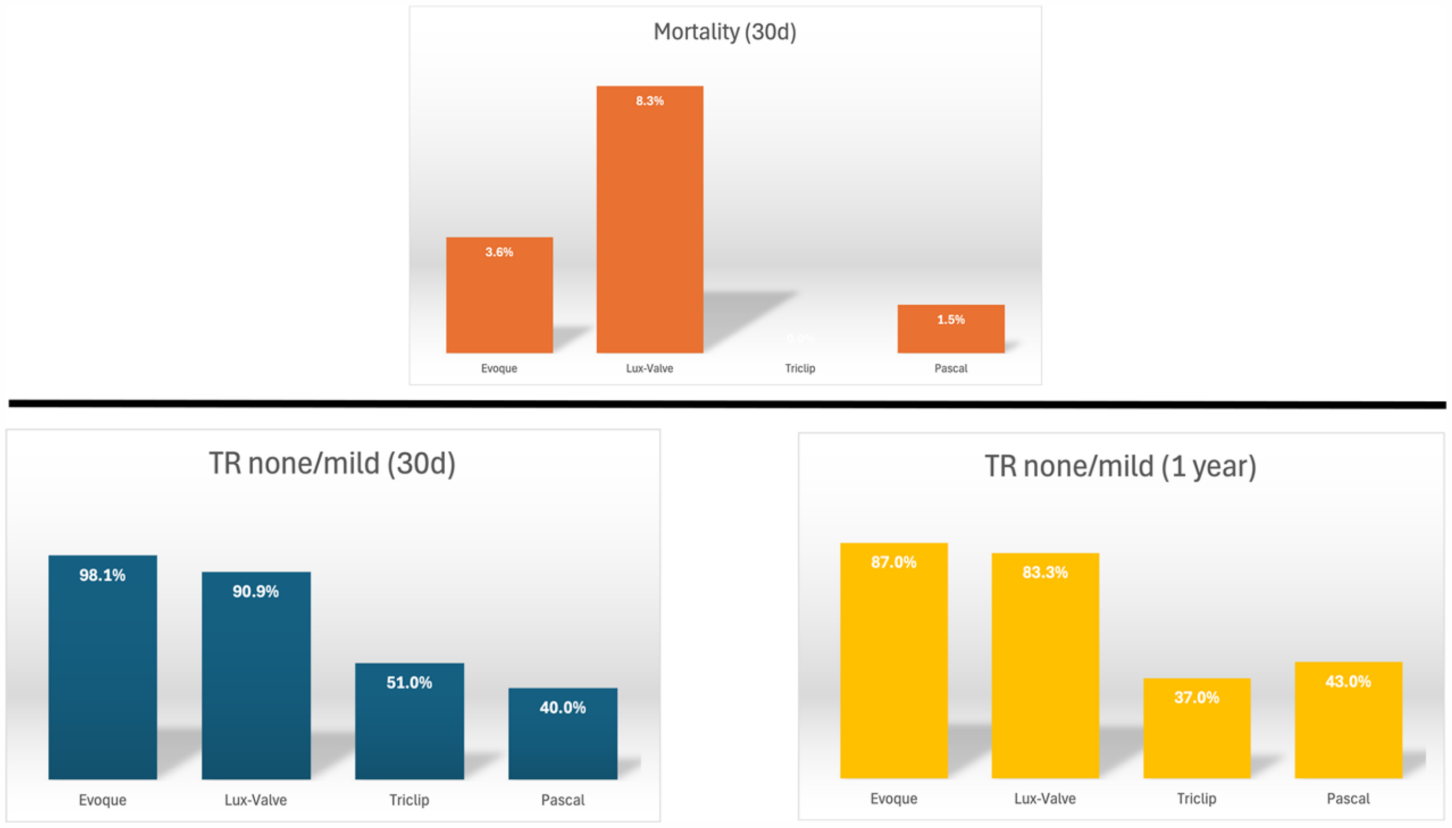
Thirty-day and one-year outcomes of transcatheter tricuspid valve intervention (20, 21, 41–45)

Clinically, secondary TR accounts for 90% of all TR cases (36). In the early stages of the disease (37) if RV dilation is not yet severe and tricuspid annular dilation occurs without significant leaflet tethering, transcatheter annuloplasty systems such as the Cardioband repair system are effective in repairing TR (38). As the disease progresses to the second stage, further dilatation of the RV and tricuspid annulus compromises leaflet coaptation, resulting in progressive leaflet tethering. At this juncture, the likelihood of achieving successful repair with an annuloplasty ring alone diminishes, necessitating a combination of edge-to-edge repair and annuloplasty (39, 40). Notably, performing TTVR at this stage may completely resolve TR. Compared to transcatheter edge-to-edge repair, TTVR can attain a mild or lesser degree of residual TR in almost all patients within 30 days and 1 year (Figure 3) (20, 21, 41–45). This resolution of TR can be maintained for up to one year, showcasing favorable functional outcomes that may positively influence long-term survival and functional status. As leaflet tethering further deteriorates in the third stage, TR escalates to massive or torrential levels, rendering repair efforts potentially futile (46).

The total GLIDE score, based on five defined variables with a maximum of 5 points (47), was associated with intraprocedural success and the procedural success endpoints of TTVr intraprocedural TR reduction by ≥2 grades and a postprocedural TR grade of moderate or less. Procedural success, defined as TTVr intraprocedural TR reduction by ≥2 grades, was achieved in 91% of patients with scores of 0 or 1 point, 47% of those with scores of 2 or 3 points, and 14% of those with scores of ≥4 points (47). A high GUILD score (≥4 points) is often indicative of advanced-stage TR, potentially accompanied by severe right heart dysfunction and pulmonary circulatory abnormalities. At this stage, treatment decisions for these patients become increasingly complex. TTVr may not achieve complete resolution of TR, and there is a risk of acute right heart failure following the procedure. Moreover, open-heart surgery is associated with a high perioperative mortality rate (48, 49).

For patients with a high GLIDE score, multidisciplinary team discussions are crucial in determining the optimal treatment plan and timepoint for the intervention, ensuring that each patient receives the most appropriate therapeutic strategy (47).

For patients with right heart dysfunction, TTVR may be considered if the RV retains a functional pump reserve. However, postoperative monitoring is essential to assess the risk of low cardiac output syndrome induced by the pop-off effect in case of pulmonary hypertension. In cases of severe RV dysfunction, both TTVR and open-heart surgery may precipitate critical right heart failure. Therefore, a thorough evaluation is necessary to determine whether prior optimization of right heart function or alternative palliative therapies should be pursued.

Apart from survival rates, improvements in quality of life are also an important consideration. In both the TRILUMINATE pivotal trial (50) and the TRISCEND II pivotal trial (51), significant quality of life benefits were observed compared to GDMT alone, and these benefits were associated with the degree of TR reduction. In the TRILUMINATE trial, improvements in Kansas City Cardiomyopathy Questionnaire Overall Summary Score scores were similar across groups, regardless of baseline TR severity. In contrast to the TRISCEND II trial, the extent of quality of life improvement was directly related to baseline TR severity, with patients with more severe baseline TR experiencing greater health status benefits (51). In addition, there were differences in the timeline of health status improvement between the two trials. In the TRILUMINATE Pivotal trial, the majority of patients showed significant improvement by 30 days post–transcatheter edge-to-edge repair. In contrast, in the TRISCEND II Pivotal trial, only moderate improvement was observed at 30 days, with continued improvement over the following six months (51). This may be due to a transient increase in RV afterload associated with TTVR.

Based on experience with mitral valve surgery, valve repair is generally prioritized over valve replacement, as conventional valve replacement surgery requires resection of subvalvular structures. This disruption to the subvalvular apparatus can damage the normal ventricular architecture, gradually leading to ventricular ’sphericalization’ and impairing ventricular function (52, 53). TTVR does not directly affect the papillary muscles and chordae tendineae; however, the implanted prosthetic valve stent may have a mild mechanical effect on adjacent tissues. In certain cases, the position of the implant may slightly alter the geometry of the RV, indirectly impacting the position and tension of the papillary muscles. Nevertheless, this effect is typically minimal and, in the vast majority of cases, does not lead to functional abnormalities.

CAVI is specifically engineered to alleviate congestion in patients with severe torrential TR who are either ineligible for surgery or present a high surgical risk. The fundamental mechanism of CAVI involves deploying a valved stent at the junction between the inferior vena cava and the right atrium to mitigate regurgitation. This intervention significantly reduces hepatic congestion, which subsequently improves hepatic and renal function, leading only to decreased symptoms of ascites and peripheral edema (54). Moreover, CAVI has the potential to enhance RV output, thereby augmenting cardiac output even though the hemodynamics do not change after TTVR. By reducing RV volume overload and gradually lowering pulmonary artery pressure, a very delayed reverse remodeling of the right heart might be induced. However, long-term follow-up with a large patient cohort is still required to confirm this supposition and thus categorize it as a palliative procedure.

Regarding safety and efficacy, the technology underpinning CAVI still requires rigorous validation through ongoing clinical trials. Furthermore, its effectiveness must be evaluated against GDMT within the framework of randomized controlled trials to establish a robust base of evidence.

### Valve durability and anticoagulation

As with transcatheter aortic or mitral valves, an inevitable issue with transcatheter tricuspid valves is their durability. Based on past experiences, the durability of bioprosthetic valves typically ranges from 10 to 15 years (55, 56). However, the durability of right-sided cardiac transcatheter bioprosthetic valved stents remains unclear. The location of the tricuspid valve makes it more susceptible to the complex hemodynamic effects within the heart, particularly in the low-pressure regions. Although the pressure in this area is relatively low, factors such as regurgitation and turbulent flow contribute to increased risks of calcification and wear. Consequently, the durability of the tricuspid valve is generally lower compared to other heart valves. Tissue-engineered bioabsorbable heart valves may offer a strategic approach having recently achieved encouraging results in the pulmonary valve domain (57). Nevertheless, further research is required in the tricuspid valve area.

Currently, there is a lack of evidence-based guidelines for antithrombotic therapy in patients undergoing transcatheter tricuspid valve interventions (58). Drawing from the experience with surgical bioprosthetic valves, in the absence of an indication for long-term oral anticoagulation (OAC), it is considered reasonable to administer vitamin K antagonists for 6 months following TTVR (59). Notably, due to the relatively lower blood flow in the right heart chambers, the risk of thrombosis in right-sided prosthetic valves is higher than in left-sided valves (60). Therefore, an extended duration of OAC may be recommended. After TTVr, single antiplatelet therapy may be considered. However, since most patients already require anticoagulation due to pre-existing atrial fibrillation, they are typically maintained on long-term OAC (61). Major bleeding is the most common serious complication following TTVI, highlighting the need for further research to determine the optimal duration of anticoagulation after transcatheter tricuspid valve intervention.

### Right ventricular dysfunction

Although transcatheter TTVR can effectively eliminate TR, a subsequent complication is the decline in RV function. Right ventricular systolic dysfunction persists 30 days post-operation, which may indicate that the mechanical function of the RV had already been impaired under chronic severe TR but was masked by the reduced afterload (62). Following TTVR, the significant reduction in TR leads to a sharp increase in afterload, thereby negatively affecting RV function.

For patients with PH, TTVR may pose several potential risks. Due to elevated pulmonary vascular resistance, the significant increase in RV afterload following TTVR may prevent the RV from adapting to the new hemodynamic state, potentially leading to low cardiac output syndrome. Additionally, the pop-off effect may further exacerbate RV afterload. Wang et al. reported the hemodynamic outcomes of the Lux-Valve procedure, finding no significant increase in pulmonary artery pressure after valve replacement (63). However, it is important to note that eliminating TR inevitably increases forward blood flow, leading to enhanced pulmonary perfusion and elevated left ventricular preload. In patients with impaired left ventricular function, this hemodynamic alteration may impose an additional burden on the heart; therefore, careful consideration is warranted when selecting TTVR for these patients.

In addition, for patients with pre-existing PH, those with moderate pulmonary artery pressure and preserved RV function may benefit from perioperative management aimed at optimizing RV function. However, TTVR is not suitable for patients with severe PH.

Sugimoto et al. (64) proposed a novel load-independent method for measuring RV contractility and found that RV dysfunction in patients with severe TR at baseline did not change after tricuspid valve surgery. While postoperative RV function can predict the outcomes of tricuspid valve surgery, the results of transcatheter devices warrant further investigation. For instance, the single-leaflet design of the Trisol valve, with its high closing volume, can mitigate the sharp increase in afterload that follows the reduction of TR.

In conclusion, while TTVR shows promise in addressing TR, careful consideration of RV function and ongoing research into device-specific impacts on afterload is essential to optimize patient outcomes.

### Transvenous leads and transcatheter tricuspid valve devices

The incidence of TR increases exponentially in patients with implanted cardiac electronic devices (65). This increase is significantly attributed to the leads passing through the tricuspid valve, which can directly interfere with the normal movement of the leaflets, preventing them from closing completely (66). Prolonged lead friction may also cause structural degeneration or damage to the tricuspid valve (67). Additionally, long-term interactions can lead to an inflammatory response, resulting in local fibrosis or scar formation, which further impairs leaflet function (68).

Endocardial leads can become trapped between the valved stent and the endocardium, resulting in transvenous lead entrapment.

In the TRISCEND trial, all nine patients with pre-existing pacemakers had their RV leads trapped by the Evoque valved stent (41). If a trans-tricuspid lead becomes trapped, it cannot be fully removed in the event of device infection, necessitating alternative surgical extraction and prolonged antibiotic therapy, both of which carry significant mortality risks. In cases of device infection, prolonged suppressive antibiotic therapy has been associated with a 25% mortality rate at one-month post-hospitalization and a 90% mortality rate at five years, with an estimated median survival of 1.43 years. Additionally, 18% of patients experience recurrence within one year (69). The need for surgical extraction also poses serious morbidity risks, particularly in the population undergoing TTVR due to high surgical risk.

In appropriate patients, percutaneous transvenous lead extraction (TLE) may be considered prior to the TTVR procedure (70). It is important to consider that in patients with TR, the lead may have become embedded or formed scar tissue due to extended presence. Removing the lead may further damage the tricuspid valve, leading to more severe regurgitation or acute valve dysfunction. In the ELECTRa registry, out of 3,555 patients who underwent TLE, 0.02%–0.59% experienced worsening of tricuspid valve function post-TLE (71). Polewczyk A et al. reported that in a study of 2,631 patients, 2.5% developed severe dysfunction following TLE. Therefore, when the risk of TLE is relatively low, performing TLE before TTVR is worth considering (72).

For patients requiring ventricular pacing, leadless pacemakers are a good option (73). However, it is advisable to implant the pacemaker before TTVR, as the delivery sheath size for devices such as Micra or Aveir is relatively large and may make the procedure more difficult (74). Utilizing a coronary sinus-based pacing system is also an option. Although studies suggest that placing transvenous leads on a bioprosthetic valve may be safe and not affect valve function, the lack of long-term follow-up necessitates caution with this approach (75). Epicardial pacing leads do not involve the valve but are associated with increased invasiveness (76).

For patients requiring an implantable cardioverter defibrillator (ICD) implant, several tricuspid-sparing options are available. Using a DF-1 lead connector, pacing/sensing leads can be implanted in the coronary sinus, while high-voltage leads can be placed in the azygos vein or coronary sinus. Completely extravascular options, such as the Boston Scientific subcutaneous ICD and the Medtronic extravascular ICD, are currently enrolling patients for clinical studies.

In summary, choosing a rhythm management method requires comprehensive consideration of the patient’s specific circumstances.

## Conclusion

TR is no longer overlooked, as it significantly contributes to cardiac morbidity and mortality. With the rapid advancement of TTVR therapies, TR can now be effectively corrected, avoiding the adverse risks associated with traditional surgery.

Compared with surgical tricuspid valve replacement, the low risk associated with TTVR devices makes them a promising therapeutic strategy. In eliminating TR, TTVR demonstrates a significant advantage over TTVr. Although some devices have received clinical approval, research on TTVR remains limited. Further studies with larger populations, longer follow-ups, and standardized management strategies are needed to advance this field.

Early feasibility studies show promising results, and ongoing research continues to explore TTVR's potential. For patients with severe TR who lack other treatment options, TTVR offers significant hope for the future.
